# House Dust Mite Induced Lung Inflammation Does Not Alter Circulating Vitamin D Levels

**DOI:** 10.1371/journal.pone.0112589

**Published:** 2014-11-12

**Authors:** Ling Chen, Kara L. Perks, Stephen M. Stick, Anthony Kicic, Alexander N. Larcombe, Graeme Zosky

**Affiliations:** 1 School of Medicine, Faculty of Health, University of Tasmania, Hobart, Tasmania, Australia; 2 Harry Perkins Institute of Medical Research, Nedlands, WA, Australia; 3 Telethon Kids Institute, University of Western Australia, Subiaco, WA, Australia; University of Lisbon, Portugal

## Abstract

Low circulating levels of 25-hydroxyvitamin D [25(OH)D] are associated with chronic lung diseases such as asthma. However, it is unclear whether vitamin D is involved in disease pathogenesis or is modified by the inflammation associated with the disease process. We hypothesized that allergic inflammation decreases the level of circulating 25(OH)D and tested this using a mice model of house dust mite (HDM) induced allergic airway inflammation. Cellular influx was measured in bronchoalvelar lavage (BAL) fluid, and allergic sensitization and 25(OH)D levels were measured in serum. Exposure to HDM caused a robust inflammatory response in the lung that was enhanced by prior influenza infection. These responses were not associated with any change in circulating levels of 25(OH)D. These data suggest that alterations in circulating 25(OH)D levels induced by Th-2 driven inflammation are unlikely to explain the cross-sectional epidemiological association between vitamin D deficiency and asthma.

## Introduction

Vitamin D is a steroid hormone that plays a key role in the regulation of innate and adaptive immunity [1]. 25-hydroxyvitamin D [25(OH)D], the major circulating form of vitamin D, is used as a marker of an individual's vitamin D status and, as such, is used to assess vitamin D deficiency [2]. 1,25-dihydroxyvitamin D [1,25(OH)_2_D], the active form, is derived from 25(OH)D, primarily in the kidneys, through enzymatic conversion by 25-hydroxyvitamin D-1α-hydroxylase (CYP27B1) [2]. CYP27B1 is also expressed by activated macrophages, dendritic cells, T lymphocytes, B lymphocytes, and respiratory epithelial cells [3]–[7]. Similarly, vitamin D receptors (VDR) are present in a range of immune cells [8] suggesting that the local production of 1,25(OH) _2_D and the up-regulation of genes with a vitamin D response element (VDRE) is a key component of the immune response.

Low serum vitamin D levels have been shown associated with chronic lung diseases such as asthma [9]–[11]. In support of the association between asthma and vitamin D deficiency, studies have also shown that VDRs and vitamin D play a role in the development of atopic and allergic disease [12], [13]. Vitamin D deficiency is common in children with mild to moderate persistent asthma and is associated with higher odds of a severe exacerbation [14]. Similarly, high vitamin D levels are associated with better lung function, less airway hyperresponsiveness and improved glucocorticoid response in severe asthmatics [10]. However, these studies have been largely cross-sectional and to date it is unclear whether vitamin D is involved in disease pathogenesis, is an indirect marker of physical activity levels or is modified by the disease process [15].

To our knowledge, no studies have reported the direct effect of allergic, Th2 driven inflammation on circulating 25(OH)D. As discussed earlier, vitamin D appears to be important in the immune response. Given that there is likely to be a local up-regulation of the conversion of 25(OH)D to 1,25(OH)_2_D during the inflammatory response, it is possible that the immediate circulating store of 25(OH)D may be depleted in settings of chronic inflammation [15]. Thus, there is a possibility that circulating vitamin D levels may be reduced as a direct result of the inflammatory response itself, which may explain the positive cross-sectional association between vitamin D deficiency and disease severity in asthma. This concept is supported by studies showing that interleukin-6 (IL-6) levels are associated with the decreased serum 25(OH)D levels in older adults [16], and the systemic inflammatory response (as indicated by c-reactive protein) has a significant effect on the plasma concentration of 25(OH)D [17]. On the basis of this possibility, we hypothesized that allergic inflammation decreases the level of circulating 25(OH)D. We tested this hypothesis using a mouse model of asthma.

## Materials and Methods

### Ethics statement

All experiments were conducted in accordance with National Health and Medical Research Council (NHMRC) animal health and welfare guidelines and were approved by the Telethon Kids Institute Animal Ethics Committee.

### Animals

Adult (8-week-old) female BALB/c mice were obtained from Animal Resources Centre (ARC, Murdoch, WA, AU) and housed in rooms with a 12∶12 hour ambient ultraviolet B free light-dark cycle, and maintained under specific pathogen-free conditions. Food (standard mouse chow), and water were provided ad libitum (Specialty Feeds, Glen Forrest, WA, AU).

### Viral priming

Mice were lightly anaesthetised with methoxylflurane (Medical Development International Ltd, Springvale, Victoria, Australia) and intranasally inoculated with 10^4.5^ plaque-forming units of influenza A/Mem/1/71 (H3N1) diluted in 50 µL of virus production serum-free medium (VP-SFM; Life Technologies, Mulgrave, Victoria, Australia). We infected mice with influenza A by pipetting small drops of solution onto the nostrils. Control mice received 50 µL of virus production serum-free medium alone (media control). Prior viral infection has been shown previously to enhance the allergic inflammation induced by subsequent exposure to allergen in mouse models [18].

### House dust mite exposure

**Figure 1:**
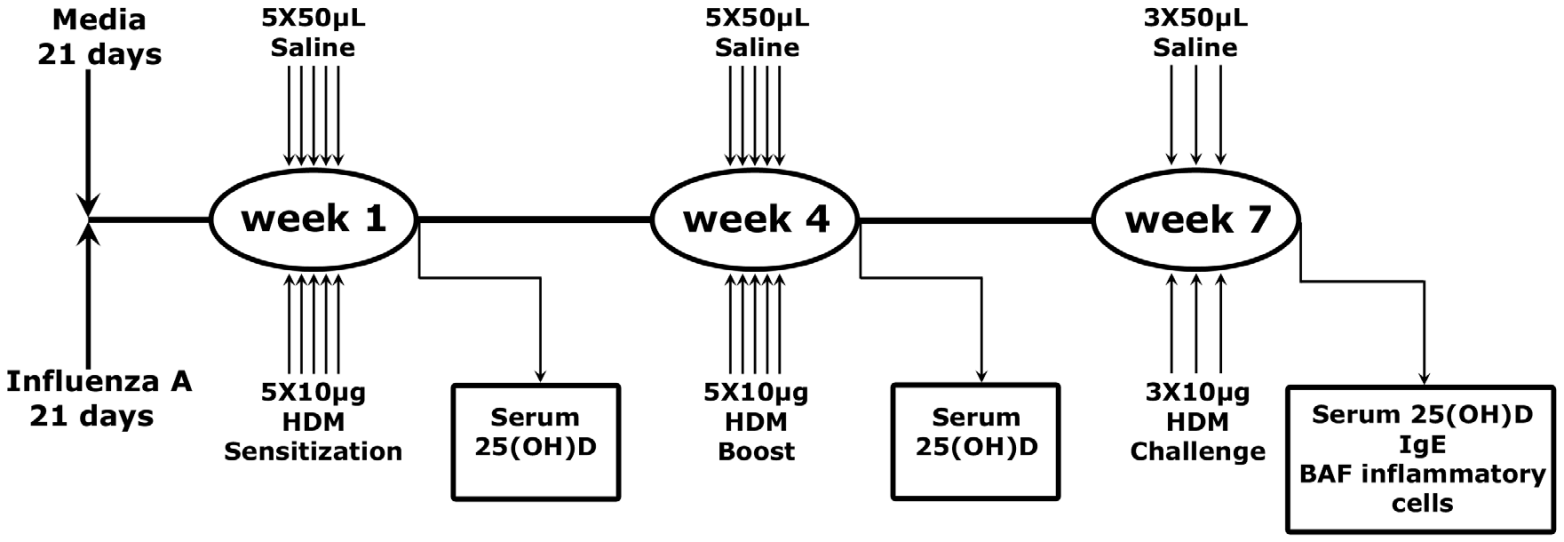
Schematic representation of the experimental protocol. Adult female BALB/c mice were infected with influenza A or media only 21 days before they were sensitized with HDM. This involved intranasal exposure to 10 µg of HDM in saline or 50µL of saline for 5 days during weeks 1 and 4, followed by intranasal exposure to 100 µg of HDM in saline or 50µL of saline for 3 days in week 7. Serum samples were collected after the last challenge of week 1, 4, and 7. BAL samples were collected after the last challenge at week 7.

21 days after influenza A infection or exposure to media (control), mice were sensitized with 10 µg of house dust mite (HDM) (dermatophagoides pteronyssinus) extract (HDM: 17.35% w/w protein, 12.47 EU/mg; Greer Laboratories, Lenoir, NC, USA) in 50 µL saline via intranasal inoculation once per day for 5 days. Mice received a “boost” (10 µg of HDM once per day for 5 days) 3 weeks later followed by a “challenge” via intranasal exposure to 100µg of HDM in saline for 3 days (Fig 1). Control mice received 50 µL saline alone (control).

### Treatment groups

The mice were randomly assigned into four treatment groups: Control (exposed to media plus saline), Flu (exposed to influenza A plus saline), HDM (exposed to media plus HDM  =  “mild” asthma), and Flu + HDM (exposed to influenza A plus HDM  =  “moderate/severe” asthma) (Fig. 1). Outcomes were measured at 24 hours after of the last HDM inoculation in week 1, 4, and 7.

### Measurement of cellular inflammation

Following euthanasia by overdose with ketamine/xylazine, bronchoalvelar lavage (BAL) fluid was collected by slowly washing 0.5 mL of saline in and out of the lungs three times via a tracheal cannula 24 hours after the last HDM challenge. The samples were centrifuged at 2000 rpm for 4 minutes and a subsample of the resuspended pellet samples was stained with trypan blue and cells were counted using a haemocytometer to obtain a total cell count. Differential cell counts were obtained by staining a subsample of the pellet on slides with Leishman's stain and counting the cells by light microscopy.

### Measurement of allergic sensitization

To assess immunoglobulin E (IgE) levels, the mice were bled via cardiac puncture 24 hours after the last HDM challenge. Serum was obtained by centrifugation of blood samples at 9300×g for 5 minutes. The supernatant was removed and stored at −80°C. The total IgE was measured by ELISA. Briefly, ELISA plates (Affymetrix eBioscience, San Diego, CA, US) were coated overnight with 2 µg/mL purified anti-mouse IgE (BD Biosciences, San Jose, CA, US). Plates were washed with 0.05% Tween 20 (Sigma-Aldrich, NSW, AU) in PBS between each step. Plates were blocked with 1% BSA in PBS before addition of purified mouse IgE isotype standard monoclonal antibody (BD Biosciences) or mouse serum. IgE was detected using biotinylated rat anti-mouse IgE (BD Biosciences) at concentration of 2 µg/mL. Streptavidin-horseradish peroxidase (GE Healthcare Life Sciences, NSW, AU) was added followed by 2,2′ –azino-bis (3-ethylbenzothiazoline-6-sulfonic acid) (Hoffmann-La Roche, Basel, Switzerland) supplemented with 0.1% hydrogen peroxide (Chem-Supply, Adelaide, AU) to produce a colour reaction. This reaction was stopped by adding 20% wt/vol SDS (Bio-Scientific, Sydney, AU). Plates were read spectrophotometri-cally at an absorbance of 405nm (Microplate AutoReader EL311; BioTek, Winooski, VT, US), and data were analysed using linear regression analysis in AssayZap (Biosoft, Cambridge, UK).

### Measurement of serum vitamin D

To assess the level of 25(OH)D, blood samples were collected via cardiac puncture 24 hours after the last HDM inoculation in week 1, 4, and 7. Serum was obtained by centrifugation of blood samples at 9300×g for 5 minutes. The supernatant was removed and stored at −80°C. A 25-hydroxyvitamin D (25(OH)D) enzyme immunoassay (Immunodiagnostic System, Boldon, UK) was performed on thawed serum samples to quantify serum levels of 25(OH)D according to the manufacturer's instructions.

### Statistical analysis

Data analysis was performed using SigmaPlot 12.5 software (Systat Software, London, UK). All the data are presented as mean ± standard deviation (SD). Two-way ANOVA plus Holm-Sidak multiple comparison tests were used to determine the statistical significance. Data were log-transformed where necessary to satisfy the model assumptions. A p-value ≤0.05 was considered significant.

## Results

### BAL fluid inflammatory cells

**Figure 2:**
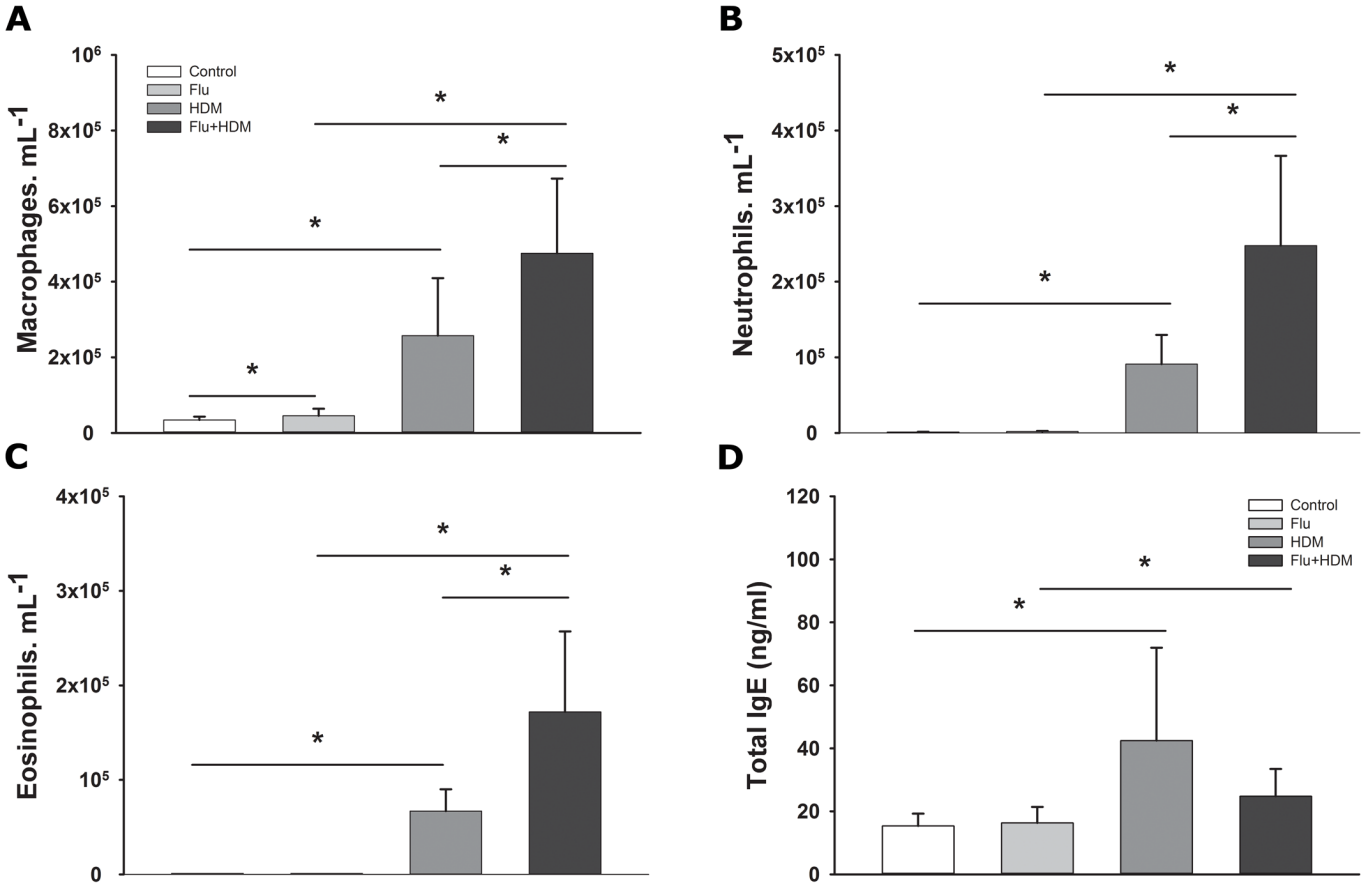
Lung inflammation. The amount of inflammatory cells (A) Macrophages, B) Neutrophils, and C) Eosinophils) of four different groups [Control (n = 9), Flu (n = 10), HDM (n = 9), and Flu + HDM (n = 9)] in BAL fluid at week 7 were counted using a haemocytometer. D) The total IgE of four different groups [Control (n = 8), Flu (n = 8), HDM (n = 8), and Flu + HDM (n = 9)] in serum at week 7 was measured using ELISA. Data are presented as mean ± SD, and were log10 transformed as appropriate to determine the statistical significance. Two-way ANOVA plus Holm-Sidak multiple comparison test was used to determine the statistical significance. *Significant differences (p<0.05).

We observed a significant effect of both influenza A and HDM on the number of macrophages (influenza A, p = 0.002; HDM, p<0.001) in the BAL fluid at week 7. There was no interaction between influenza A and HDM suggesting that effects were additive (Fig. 2A). Interestingly, while influenza A alone had no effect on the levels of neutrophils (p = 0.11) exposure to influenza A prior to HDM sensitisation significantly enhanced (p<0.001) the neutrophil influx induced by HDM (p<0.001). Similarly, influenza A alone did not alter eosinophil levels (p = 0.99) but did enhance (p<0.001) the eosinophilia induced by HDM exposure (p<0.001) (Fig. 2B, 2C).

### Serum total IgE

Exposure to HDM significantly increased serum total IgE at week 7 (p = 0.003) (Fig. 2D). This effect was not modified by exposure to influenza A (p = 0.13) (Fig. 2D).

### Serum vitamin D

**Figure 3:**
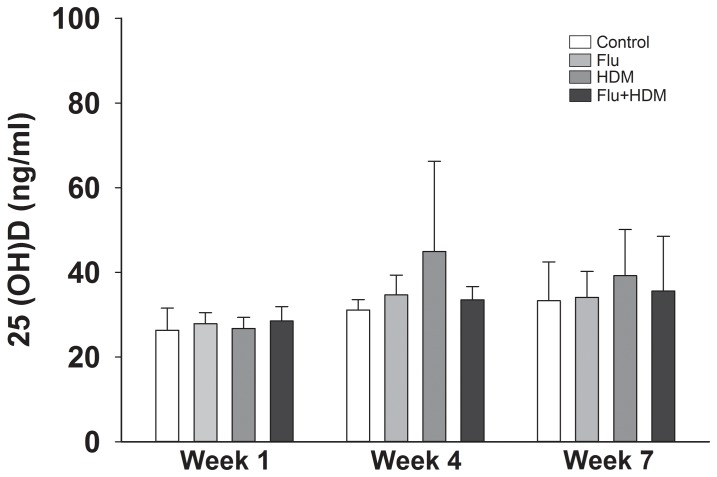
Serum vitamin D levels. Serum 25(OH)D levels of adult female BALB/c mice were measured using ELISA. Serum samples were from four different groups and collected at week 1 [Control (n = 5), Flu (n = 5), HDM (n = 5), and Flu + HDM (n = 5)], 4 [Control (n = 5), Flu (n = 5), HDM (n = 5), and Flu + HDM (n = 5)], and 7 [Control (n = 10), Flu (n = 10), HDM (n = 10), and Flu + HDM (n = 10)]. Data are presented as mean±SD. Two-way ANOVA plus Holm-Sidak multiple comparison test was used to determine the statistical significance.

We observed no significant effects of influenza A infection or HDM exposure on serum 25(OH)D levels at week 1 (Flu, p = 0.32; HDM, p = 0.74), week 4 (Flu, p = 0.44; HDM, p = 0.22), or week 7 (Flu, p = 0.66; HDM, p = 0.25) (Fig. 3).

## Discussion

To test if allergic inflammation reduces the circulating levels of 25(OH)D, we used a HDM induced mouse model of allergic airway inflammation. In mice, when given via the airway, HDM induces a substantial inflammatory response. By exposing the mice to influenza infection prior to allergic sensitization we were able to induce a more severe inflammatory response; a phenotype which is more consistent with severe, steroid resistant asthma [18]. Despite the level of inflammation we induced, there was no effect on circulating 25(OH)D in these mice. This is contrary to our hypothesis and suggests that Th-2 driven inflammation has no impact on circulating levels of 25(OH)D. The observation that CYP27B1 is expressed in the lungs of both mice [19] and human [7] suggests that the association between low serum vitamin D levels and asthma in human is unlikely to be explained by conversion of 25(OH)D to 1,25(OH)_2_D.

T-helper type 2 (Th2) cells play an important role in the pathogenesis of allergic diseases including allergic asthma [20]. In line with this, HDM exposure in mouse models is associated with the production of Th2 cytokines [21]. Given the importance of 1,25(OH)_2_D in modulating T cells responses [22], we expected Th2 driven inflammation to reduce circulating 25(OH)D levels; however, this was not the case. The reaction of immunoglobulin E (IgE) with its receptors is central to the allergic response [23]. IgE-dependent mast cell activation plays an important role in allergic airway inflammation by recruiting Th2 cells into the site of allergic inflammation [24]. Ovalbumin (OVA) is frequently used allergen that induces a robust, allergic pulmonary inflammation in laboratory rodents. However, OVA is not a relevant aero-allergen in human asthma, and other groups have used alternative allergens that may have greater clinical relevance, such as HDM and cockroach extracts [25]. For this reason we chose to focus on HDM in this study. However, it should be pointed out that levels of IgE in the serum were low in our HDM-sensitized mice model compared to other mouse models of allergic airway inflammation [26]. This poor antibody response is characteristic of the HDM mouse model [27] and represents a potential limitation. However, we were able to induce a robust inflammatory response, which included a significant influx of eosiniphils, in the mice allowing us to test the effect of inflammation on circulating 25(OH)D levels. Our model also showed a robust neutrophilia that was equivalent to the level of eosinophils. This is often observed in the HDM model [28] and is consistent with the diversity of inflammatory cell profiles that is observed in human asthmatics [29].

We should also acknowledge that it is well known that vitamin D is important in Th1 inflammation [30] and, as such, we were not able to determine whether 25(OH)D levels are altered by others forms of inflammation. It should also be noted that the mice were receiving constant vitamin D through their diet. Vitamin D from dietary sources is incorporated into chylomicrons and transported by the lymphatic system into the venous circulation [31]. In our mouse model, this ongoing supply of vitamin D is likely to have replenished the store of 25(OH)D which may have masked any local decreases in circulating 25(OH)D caused by conversion of 25(OH)D to 1,25(OH)_2_D.

In this study we were able to show that HDM driven lung inflammation has no impact on circulating 25(OH)D levels. This was in the setting of otherwise healthy mice that were receiving a constant supply of vitamin D through their diet (2000IU/kg of feed; Specialty Feeds Mouse Cubes, Glen Forrest, Western Australia) which is enough to produce serum levels of 25(OH)D that are consistent with sufficiency in humans [32]. This suggests that a constant supply of vitamin D is able to prevent the depletion of 25(OH)D as a result of conversion to 1,25(OH)_2_D. This eliminates one possible explanation for the cross sectional association between serum vitamin D levels and asthma severity.
